# Evaluation of the communication strategy for promoting physical activity in a cross-company network in Germany: A mixed-methods analysis

**DOI:** 10.3389/fpubh.2022.905451

**Published:** 2022-12-15

**Authors:** Carina Hoffmann, Andrea Schaller

**Affiliations:** ^1^Working Group Physical Activity-Related Prevention Research, Institute of Movement Therapy and Movement-Oriented Prevention and Rehabilitation, German Sport University Cologne, Cologne, Germany; ^2^Department Research and Development, Institute for Occupational Health Promotion, Cologne, Germany

**Keywords:** workplace health promotion, physical activity, health communication, strategies, mixed-methods, cross-company networks

## Abstract

**Introduction:**

The workplace is considered a promising setting for reaching physically inactive adults, but participation quotes in workplace health promotion (WHP) remain low. Regarding the low participation in WHP, the question emerges concerning the importance of health communication strategies. This paper presents the results from the evaluation of the communication strategy of a cross-company network for promoting physical activity and derives findings for the successful communication of measures.

**Materials and methods:**

Quantitative and qualitative data sources were used to evaluate the communication strategy. The methods applied included individual semi-structured interviews (*n* = 14) and the monitoring of the usage of digital communication channels.

**Results:**

The analysis revealed that the usage of the digital communication channels within this study was subjected to major fluctuations and a variety of factors must be considered when communicating physical activity measures in a cross-company network. It is important to engage in appropriate communication management that explicitly takes the interpersonal communication and the organizational circumstances into account.

**Conclusion:**

This study revealed which factors may have an influence on the successful communication of physical activity measures in the context of WHP in cross-company networks. Thus, it makes an important contribution to the transfer of science and practice as it captured relevant questions from the field of WHP.

**Trial registration:**

German Clinical Trials Register (DRKS)-ID: DRKS00020956; Date of registration: 18 June 2020, https://drks.de/search/de/trial/DRKS00020956.

## Introduction

The importance of promoting physical activity in the population is undisputed (1) and the workplace is considered a promising setting for reaching physically inactive adults (2). Overall, workplace health promotion (WHP) and occupational safety are becoming increasingly important (3). Being it due to aging workforce or influences such as the coronavirus pandemic (3). To promote the health of employees, companies in Germany have the option of offering behavior- and environmental-related measures such as courses, information campaigns, or consulting services, supported by the statutory health insurance funds (4–6). Mostly, WHP measures are complex interventions in complex settings (7, 8) and especially, the implementation of organizational WHP measures implicates challenges (9).

In the pre-coronavirus year 2019, health insurance funds spent about 240 million euros on implementing such measures in German companies, representing an increase of 164 million euros compared to 2015 (10, 11). Moreover, the number of employees reached increased from 1.3 million in 2015 to 2.3 million employees in 2019 (10, 11). Thereby, most of the behavioral-related measures implemented in German companies focused on physical activity. In the context of environmental-related measures, interventions relevant to physical activity ranked second behind measures for the health-promoting design of work activities and conditions (10).

Despite the increased expenditure of health insurance funds and the number of employees reached, it is currently assumed that only 7% of all employees subjected to social insurance contributions in Germany were reached with WHP, in 2019 (10, 12).^1^ Additionally, previous research has indicated that the participation quotes in WHP measures are still low (13, 14) or even declining (15). Thereby, the reasons for non-participation can be manifold (13, 16–20). According to Nöhammer et al. (17) and Walter et al. (21), suitable information and strategic planning of the communication process seem to be important for participation in WHP measures.

Regarding the low participation in WHP, the question emerges concerning the importance of health communication strategies. According to Baumann and Hurrelmann (22), health communication refers to “the conveying and exchange of knowledge, experiences, opinions and feelings that are related to health or illness, prevention or the health care process, the health economy or health policy […]” [22, p. 13]. Thereby, from the authors' perspective, communication can take place on an interpersonal, organizational, or societal level and can be direct-personal or mediated by the media (22). Specifically, digital health communication has increased significantly in recent years and enables new potential for health promotion (23–25). It also plays a major role in the physical activity communication (26). Thereby, low usage data and high attrition rates are still a common problem (27, 28). Basically, health communication can be seen as a growing and complex interdisciplinary field of research that includes, among others, communication science, psychology, sociology, medicine, or social marketing (22, 29–31). Therefore, an examination of this subject area is conceivable based on a variety of theories as well as approaches from the most diverse research fields (31). However, research on health communication needs to be expanded (31), especially in the field of prevention and health promotion (32).

In order to raise people's awareness about the importance of health, many communication campaigns have been implemented in recent years in Germany (33). For example, the Exercise (Trimm)—campaign of the German Sports Association, aimed at promoting the physical activity behavior of major demographic groups (34). Overall, research has found medium evidence of mass-media campaigns in the context of physical activity promotion (2, 35–38). Thereby, the importance of an evidence-based conception and systematic implementation as well as the target group-oriented design of campaigns is highlighted (39–41). As Bonfadelli and Friemel (39) noted, campaigns can pursue three strategies to achieve their goals: a cognitive strategy, an affective strategy, and a social strategy. Ideally, campaigns should address all the levels in order to accentuate different motives and gratifications (39). The literature (39, 41) emphasizes that the consideration of theoretical models and theories of communication is crucial for the successful planning and realization of health communication campaigns. There are a large number of models that provide explanations for the attention paid to campaign messages, how they are processed, and how they influence the behavior of the target group (33, 39). In the context of behavior change, this includes, for example, the Transtheoretical Model (42), which makes it possible to define goals, target groups, and messages depending on the stage of behavior change (39). Referring to the communication theories, the Elaboration-Likelihood-Model (43) has practical relevance, since it postulates that the influence of media-mediated messages can vary depending on the situation (39, 41).

Basically, there is a large body of literature concerning theories on the development and evaluation of communication campaigns [cf. (39)]. Nevertheless, less attention has been paid to a systematic health communication approach in the workplace setting (44), and hardly, no theories exist to date on the systematic use of communication tools in WHP (21). With their model for the systematization of communication tools, Walter et al. (21) provided an initial approach for practitioners in WHP on how communication tools can be used in a targeted manner. As Faller (45) has noted, the aims of communication in WHP are to announce the measures, increase the employees' knowledge about health, and motivate them to participate. Thus, the goals of health communication in WHP are comparable to those of health marketing (45), which is based on a systemic approach (45–47). However, from the perspective of the employees, information about WHP measures in the company is often insufficient or not comprehensible enough (48, 49), so that additional research is required to explore this research domain in more detail.

The present study was part of the model project “KomRueBer,” which aimed to conceive, implement, and evaluate a cross-company network offering a multi-component intervention promoting physical activity of employees in small- and medium-sized companies (50, 51). The study was composed of two parts, with the first part focusing on the development of the cross-company network and a multicomponent intervention for promoting physical activity (50). Subsequently, the multicomponent intervention was implemented and evaluated, with the evaluation based on an impact model (logic model) and a focus on process evaluation (51). In Germany, the establishment of cross-company networks is an acknowledged approach to specifically support health promotion for employees in small companies (6, 50). The literature (52) recommends a consolidation of four up to twelve companies with a total of at least 100 employees. The organizational and administrative effort increases with the size of the network (52), which consequently also applies to the communication processes. Basically, communication is a challenge in networks, but it contributes to the success if it is done professionally (53). To the authors' knowledge, to date, no studies have been conducted on the communication of physical activity measures in such cross-company networks.

The aim of the present paper was to present the results from the evaluation of the communication strategy of the cross-company network promoting physical activity. It is intended to contribute to the output level in the context of the impact model-based evaluation of the KomRueBer study. Given the importance of strategic planning of communication processes for participation in WHP and on the other hand, still limited research concerning systematic approaches to health communication in the workplace setting, this article also aims to provide important insights on how employees can be systematically informed about physical activity measures. Particularly, in the context of cross-company networking, there is a serious research gap, in which this article should contribute to clarify. The related research questions were as follows: (1) How is the usage of the digital communication channels (media) within the communication strategy to promote physical activity in the cross-company network? (2) How do the stakeholders of the cross-company network assess the communication strategy for promoting physical activity? (3) What are the facilitating factors for and barriers to successful communication of physical activity measures in the cross-company network?

## Materials and methods

The study was conducted in a technology park in Germany with about 90 companies and an estimated 2,000 employees. In total, seven companies formed the core of the cross-company network and actively participated in the KomRueBer project. Ethical clearance for the KomRueBer project was obtained from the Ethics Committee of the German Sport University Cologne (reference number: 068/2020). The study was conducted in compliance with the Declaration of Helsinki and is registered in the German Clinical Trials Register (DRKS00020956). A mixed-methods design was applied to evaluate the communication strategy. We choose this approach to develop a better overall interpretation of the communication strategy. Data for the present evaluation were gathered from April 2020 to September 2021.

### The KomRueBer communication strategy

The KomRueBer communication strategy comprised the systematic development of messages and communication channels for promoting physical activity measures and their dissemination on site. Likewise, the communication strategy included the entire communication management to make the measures and the project known. The messages and communication channels were developed by an agency in collaboration with the KomRueBer project team and conveyed three messages in terms of content:

- Message 1: “We create movement. Any kind of movement is good. It makes you stronger, it makes you more self-confident, it is fun! We enable movement in your workday: As an employee in the technology park (name of the town) we—together with our project partners—are creating simple proposals, which will pick you up from where you are. Movement which motivates you.”
- Message 2: “We are many. And we are determined to champion all the good that movement brings. Within a partner network, with loads of ideas, proposals and joy, we are committed to encourage movement for the many. We are on your side. You are one of us. You are in the right place.”
- Message 3 / Slogan: “There is movement in this.”

Table 1 shows the communication channels used to disseminate the messages and information about the project and its measures.

**Table 1:** Communication channels within the KomRueBer project.

**Communication channel (media)**	**Type of media**	**Application area**
Posters	Print	Announcement of the project and measures on site
Business cards with contact name of the project	Print	Distribution at on-site events so that employees have a direct point of contact for the measures and project
Banner	Print	Drawing attention to the project at events on site
Website	Digital	Central information and registration platform for the physical activity measures
Newsletter tool	Digital	Personalized e-mail marketing to inform about new measures

**Figure 1:**
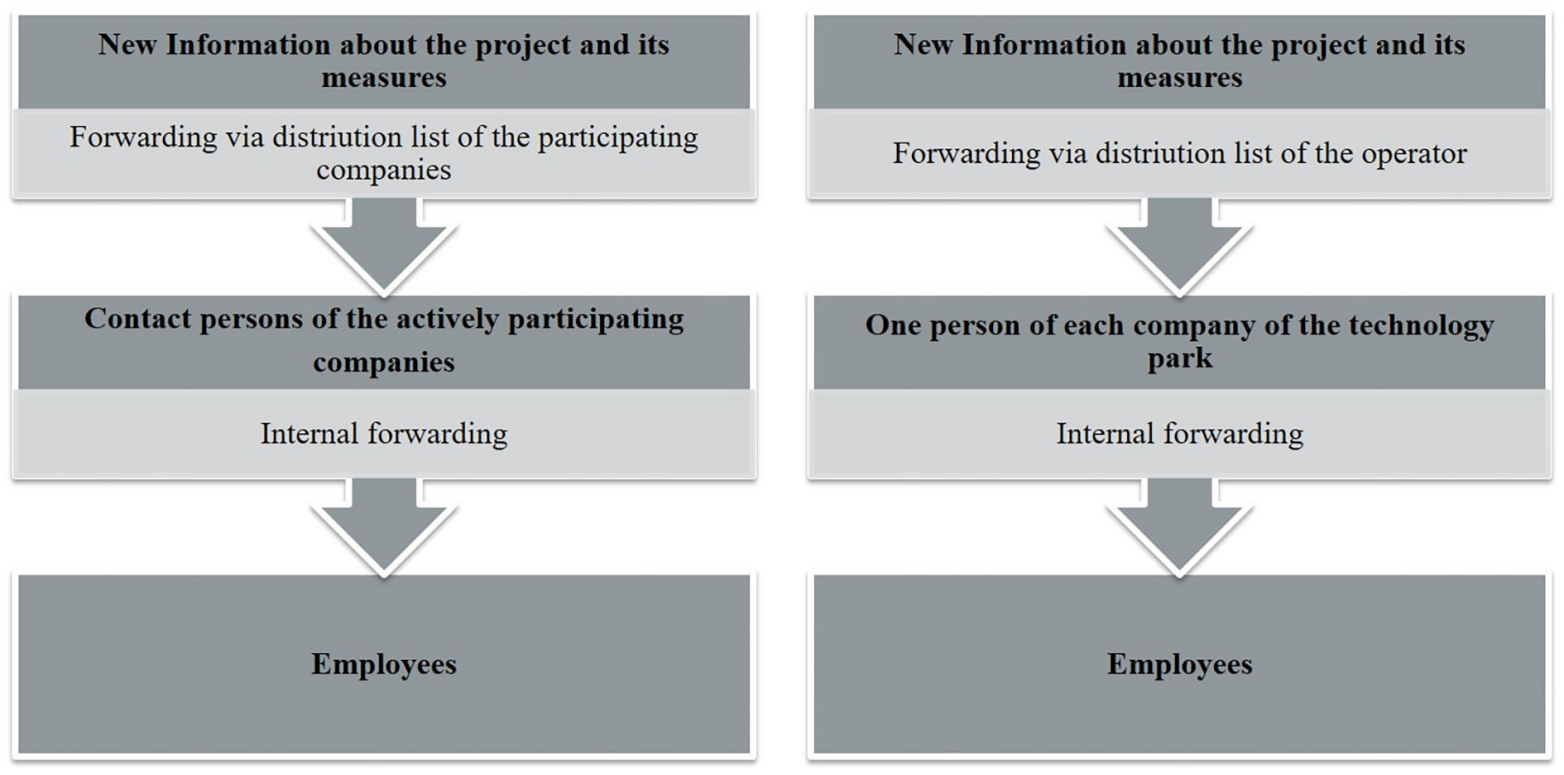
Communication paths for the dissemination of information.

The KomRueBer communication strategy addressed employees on site, contact persons of the companies collaborating and not collaborating in the KomRueBer project, and the management of the technology park. Employees could seek information by registering for the newsletter or visiting the website, where all physical activity measures were presented. Likewise, the project was advertised on posters on site. Figure 1 illustrates the communication paths for the dissemination of information in the cross-company network. We defined communication paths as the forwarding of information about the project and its measures *via* company representatives to their employees. In total, two supplementary communication paths were used for the dissemination of information.

On the one hand, new information was sent from the project team to the contact persons (generally one to four persons of each company) of the actively-participating companies (distribution list of the participating companies), while on the other hand, a distribution list of the operator of the technology park—which was addressed to all companies on site (generally addressed to one contact person)—could be used occasionally. This also made it possible to reach companies and their employees that were not directly involved in the KomRueBer project. The recipients were asked to forward the information to the employees in the respective company.

### Quantitative study design

To answer research question 1 (How is the usage of the digital communication channels of the communication strategy to promote physical activity in the cross-company network?), the number of users of the website (people who interacted with the website) and the number of subscribers of the newsletter were monitored. Usage data of the website were collected after consent to cookie use based on google analytics. The number of users was monitored weekly from 6 April 2020 (week 1; release of the website) to 22 August 2021 (week 72). In addition, the number of physical activity measures offered, project management activities (e.g., dispatch newsletters), or external factors (e.g., imposition of coronavirus-lockdown, holiday period) were assessed. The newsletter subscribers were recorded by the program Sendinblue (Sendinblue GmbH, Berlin, Germany). The subscription was at no charge and it was necessary to agree to the applicable privacy policy prior to subscribing. The number of subscribers was gathered between 25 June 2020 and 19 July 2021. For this purpose, the total number of recipients was recorded for each week. Monitoring data were displayed descriptively [frequency (n), mean (mean), standard deviation (±SD)].

### Qualitative study design

Semi-structured interviews were conducted to answer research questions 2 (How do the stakeholders of the cross-company network assess the communication strategy for promoting physical activity?) and 3 (What are the facilitating factors for and barriers to the successful communication of physical activity measures in the cross-company network?). The first author (C.H; PhD. candidate and trained in qualitative research) conducted the semi-structured interviews from August to September 2021. The researcher and interviewee knew each other through the project. Due to the prolonged pandemic situation, the interviews were realized by video conference. The interviews were conducted in German, anonymized using a code and digitally recorded. During the interviews, pictures of the media and the messages were displayed, so that the participants could regard them calmly again. Handwritten field notes—taken during and after the interviews—completed the data collection. Demographic data were collected after the interview has been completed.

#### Sample

The study population comprised different stakeholders in the project (exercise providers, company representatives, network partners from public, economy, and society/politics). The recruitment was conducted through the first author *via* mail or telephone. Each of the project's 18 stakeholders was asked if they were interested in participating. In total, fourteen of them expressed their willingness to participate. No feedback was received from four stakeholders. Informed consent was taken from each participant before the interview was conducted. In total, four male and ten female stakeholders participated in the interviews. The sample comprised two company representatives, eight network partners, and four exercise providers. In total, four participants had a double role (one network partner and exercise provider; three company representatives and network partner). The age of the participants ranged between 33 and 60 (mean 47 ± 8). The interviews lasted from 35 to 70 min, with an average interview duration of 50 min (±11).

#### Interview guide

The conception of the interview guide was based on the McGuire's model of persuasion (54). In addition, the results of the monitoring of the usage data of the digital communication channels were incorporated into the development of the interview guide. The questions were collected collectively in the project team and the interview guide pilot was tested internally. Table 2 shows the topics and key questions. Supplemental questions were used to support the conversation. The order of the questions was adapted flexibly to the course of the interview, if necessary.

**Table 2:** Topics and key questions of the interview guide (translated from German).

**Topic**	**Key question(s)**
Existence of appropriate framework conditions to be exposed to the messages and the project	“From your point of view: how would the ideal announcement of this project have looked in this cross-company setting without Corona?”; “How do you rate the access routes?”; “How do you assess the communication channels for the target group of employees in a cross-company environment?”
Structuring of cross-company communication including facilitating factors and barriers	“From your point of view, how would the ideal cross-company communication be structured?”
Evaluation of messages and media in terms of their generation of attention, arousing interest, and comprehension.	“How successfully does the design of the messages catch your attention?”; “How strongly do the statements personally affect you?”
Overall evaluation of the communication strategy	“What do you think much does this communication strategy motivate movement among the present employees?”; “In hindsight, how do you evaluate the amount of information within the framework of the project?”

#### Transcription and data analysis

The transcription was conducted according to the rules of Dresing and Pehl (55). Each transcript was double-checked, whereby the interviews were analyzed by two researchers according to structured content analysis (56). Latter is comparable to the framework method (57). Based on the interview guide, the main categories were formulated deductively. In addition, main categories were derived from the text material inductively. Subsequently, the text material was assigned to the main categories before sub-categories were determined inductively in the next step. The interviews were analyzed using MAXQDA 20 software (VERBI GmbH, Berlin, Germany).

## Results

### Usage of the digital communication channels

**Figure 2:**
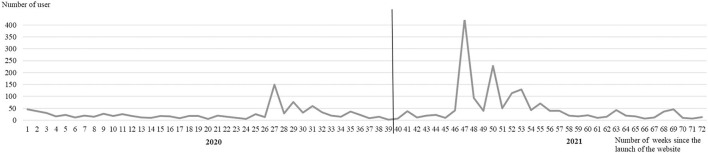
Website users per week over time.

Figure 2 shows the website users per week over time. The average number of users per week was 37 (±58.8), ranging between 425 (week 47) and 2 users/week (week 39).

**Figure 3:**
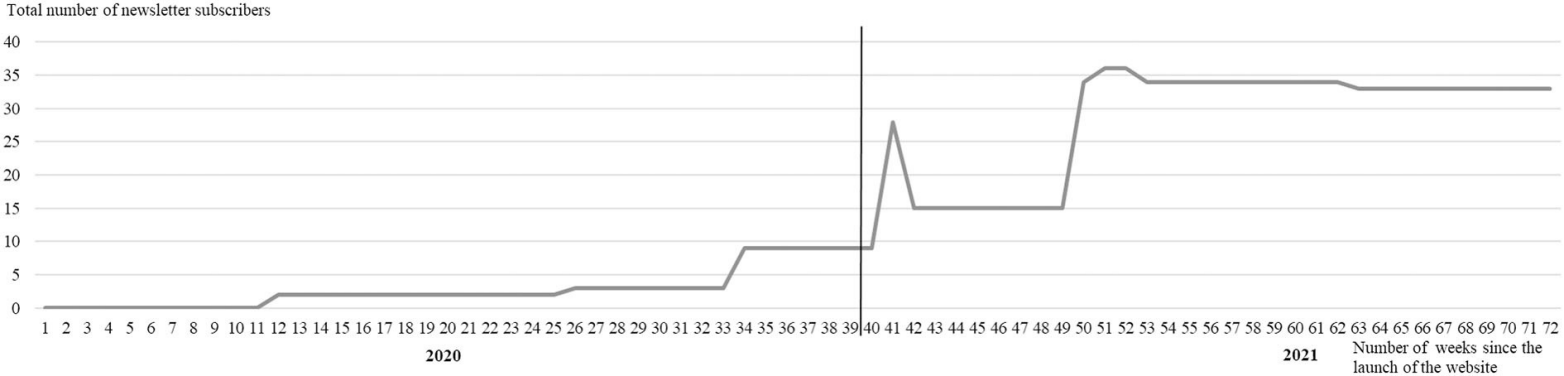
Total number of newsletter subscribers over time. Note: 0 between week 1 and 11 caused to later starting of newsletter dispatch compared to activation of the website.

Figure 3 illustrates the total number of newsletter subscribers over time. The maximum number of subscribers of the newsletter was recorded in weeks 51 and 52 with 36 persons. The first newsletter subscribers were registered in week 12 (2). Since week 26 three subscribers and since week 34 nine subscribers were recorded. In week 41, the number of subscribers increased up to 28 and dropped again to 15 subscribers within 1 week. An increase to 34 subscribers was registered in week 50 and to 36 subscribers in week 51. From week 53 to week 62, the number of subscribers was again 34, before declining again to 33 persons after week 63. Thus, the highest number of new registrations (19) was in project weeks 41 and 51, whereby the highest number of unsubscriptions (13) was in project week 42.

Table 3 shows the project activities in the same period and external factors (e.g., holiday periods, lockdown periods due to coronavirus pandemic). Only those weeks in which project activities took place or external factors were recorded are listed. The distribution list of the operator of the technology park was used three times to inform about new physical activity measures for the cross-company network (weeks 9, 18, and 27). In addition, the company representatives of the companies actively participating in the project were informed eighteen times about new physical activity measures (weeks 1, 7, 8, 12, 16, 26, 30, 31, 35, 41, 46, 50, 54, 55, 57, 63, 68, and 69). Overall, twelve newsletters were dispatched (weeks 12, 26, 35, 41, 46, 50, 54, 55, 57, 63, 68, and 69) and three steering groups of the KomRueBer project were implemented (weeks 29, 43, and 62). Besides, the project was presented at one company meeting (week 32) and within a course measure (week 49). Social media was used once by an exercise provider to inform about the measures (week 47).

**Table 3:** Project and communication management and external factors in the course of the project.

**Month**	**Project week**	**Project and communication management**	**External factors**
April	1	Dispatch of project start flier *via* mail to company representatives	First lockdown since mid-March; School vacations
	2		School vacations
	4		Mandatory wearing of masks
May	6		Relaxation of coronavirus lockdown
	7	Reference to project website *via* mail to company representatives	
	8	Announcement of new measures *via* mail to company representatives	Further relaxation of coronavirus lockdown
June	9	Announcement of new measures *via* distribution list of the operator	
	11		Opening of canteens and cafeterias
	12	Announcement of new measures *via* mail to company representatives; Dispatch of the first newsletter	
July	13		School vacations
	14		School vacations
	15		Further relaxation of coronavirus lockdown; School vacations
	16	Mail to company representatives with further information about the recent measures	School vacations
	17		School vacations
August	18	Announcement of new measures *via* distribution list of the operator	School vacations
September	26	Announcement of new measures *via* mail to company representatives; Dispatch of the second newsletter	
October	27	Announcement of new measures *via* distribution list of the operator	
	28		School vacations
	29	Steering meeting with the company representatives	School vacations
	30	Mail to company representatives with further information about the recent measures	End of October; appeal for home office working
November	31	Mail to company representatives with further information about the recent measures	Partial coronavirus lockdown; among others closure of fitness studios
	32	Presentation of the project in a works meeting within one company	
December	35	Announcement of new measures *via* mail to company representatives; Dispatch of the third newsletter	
	38		School vacations
	39		School vacations
**2021**			
January	41	Announcement of new measures *via* mail to company representatives; Dispatch of the fourth newsletter	Beginning of January; tightening of the coronavirus lockdown
	43	Steering meeting with exercise provider	End of January; mandatory home office working
February	46	Announcement of new measures *via* mail to company representatives; Dispatch of the fifth newsletter	Extension of coronavirus lockdown
	47	Instagram post about an exercise provider's measure	
March	49	Presentation of the project within the framework of one measure	
	50	Announcement of new measures *via* mail to company representatives; Dispatch of the sixth newsletter	
April	52		School vacations
	53		School vacations
	54	Announcement of new measures *via* mail to company representatives; Dispatch of the seventh newsletter	
	55	Announcement of new measures *via* mail to company representatives; Dispatch of the eight newsletter	
May	57	Announcement of new measures *via* mail to company representatives; Dispatch of the ninth newsletter	
June	62	Steering meeting with the company representatives	Beginning of June; reopening of fitness studios
	63	Announcement of new measures *via* mail to company representatives; Dispatch of the tenth newsletter	End of June; end of mandatory home office working
July	67		School vacations
	68	Announcement of new measures *via* mail to company representatives; Dispatch of the eleventh newsletter	School vacations
	69	Announcement of new measures *via* mail to company representatives; Dispatch of the twelfth newsletter	School vacations
August	70		School vacations
	71		School vacations
	72		School vacations

### Appraisal of the communication strategy from the stakeholders' perspective

Concerning research question (2), five main categories were identified: *(a) communication paths, (b) communication channels, (c) design of the media, (d) messages, and (e) overall communication strategy*. Table 4 shows the main categories, their definitions, the related sub-categories and characteristics.

**Table 4:** Coding system: appraisal of the communication strategy.

**Main category (definition)**	**Sub-categories**	**Characteristics**
Communication paths (Forwarding of information about the project and its measures *via* company representatives to their employees; distribution list of the participating companies and the operator)	Advantages	• Established communication paths • Wide range • Low costs • Concrete contact persons
	Disadvantages	• Interface problem • Information flooding • Unclear accessibility of employees
	Improvement Suggestions	
Communication Channels (Media used to convey the messages and the information about the measures to the employees in the cross-company network; newsletter, website, posters)	General appraisal	
	Appraisal of the newsletter	• Advantages • Disadvantages
	Appraisal of the website	• Advantages • Disadvantages
	Appraisal of the posters	• General appraisal
Design of the media (Style of the communication channels and the logo)	Generating attention	• Positive appraisal • Negative appraisal
	Design of the poster	• Picture material • Color selection • Suggestions for improvement
	Design of the website	• Amount of information • Visual effect
	Design of the logo	• Physical activity is reflected in design • Draws interest • High memorability • Expressive • Too text-heavy • Missing expressiveness • Design unimportant
Messages (Key statements about the KomRueBer project)	Message 1	• Effect • Improvement suggestion • Information content
	Message 2	• Effect • Information content • Improvement suggestion
	Slogan	• Effect • Improvement suggestion
Overall communication strategy (Overall concept of the communication strategy)	Contribution to promoting physical activity on site Amount of information General appraisal	• Can contribute • Cannot contribute • No assessment possible

Within the main category “*communication paths,” three* sub-categories could be defined: “advantages,” “disadvantages,” and “improvement suggestions.” Advantages comprise positive aspects of the distribution lists (e.g., established, low costs, and firm contact persons). As disadvantages (unfavorable aspects of use of the communication paths), interface problems, potential information flooding, and uncertainty regarding the degree to which the target group has been reached were mentioned. Suggestions for improvement—meaning ideas to optimize the communication paths—included extending the list to several representatives of one company and establishing a separate sender, so that information could be directly assigned by the recipients.

“*Communication channels”* was divided into four sub-categories. The “general appraisal” was that the combination of poster, website, and newsletter is useful. For the sub-categories of “appraisal of the newsletter” and “appraisal of the website,” the interviewees again mentioned advantages and disadvantages. As advantages of the newsletter, the accessibility of the interested employees and the reminder functionality were named, whereas the need for an active registration, flood of e-mails, an e-mail address as a prerequisite, and the possibly limited up-to-dateness were mentioned as disadvantages. Named advantages of the website were the continuous information and the up-to-dateness while the need for an active access of the employees was assumed as a disadvantage. Regarding the “appraisal of the poster,” they were seen as a useful way to draw attention to the project and its measures. It was highlighted that these should be used more intensively.

“*Design of the media”* comprised the sub-categories “generating attention,” “design of the poster,” “design of the website,” and “design of the logo.” In the sub-category “generating attention” (extent to which the design of the media attracts the attention of the interviewees), both positive and negative statements were given, whereby the majority of stakeholders expressed positive statements. The sub-category of “design of the poster” (statements concerning the appearance of the poster) showed the two characteristics of picture material (e.g., lack of expressiveness, good image variety) and color selection (e.g., too dismal, fresh). Besides, the stakeholders made suggestions for improvements, such as making reference to specific measures on the posters. Concerning the sub-category “design of the website” (statements about the processing of the information platform), stakeholders made reference to the amount of information represented (e.g., too much information, good overview) and the visual effects (e.g., too confusing, underlines topic of physical activity). Feedback concerning the “design of the logo” (composition of the project logo) showed a wide range of characteristics. However, in the majority of cases, it became clear that the design of the logo reflects the topic of physical activity well. Overall, it was noticeable that the feedback on media design covered a broad spectrum with various opinions.

Within the main category “*messages,”* three sub-categories were identified. Concerning the sub-categories of “message 1” and “message 2” (statements that related to the evaluation of the respective formulations), a considerable diversity of opinions was registered which could be classified into the characteristics of “effect,” “information content,” and “improvement suggestion.” The stakeholders mentioned a wide range of effects (e.g., underlining that physical activity is good, clarifying that everyone is welcome) from which no uniform propensity could be derived. Opinions also differed regarding the information content of the messages (e.g., sufficient, too much text). Some improvement suggestions (e.g., simpler language, addressing the target group more specifically) were stated. The sub-category of “message 3/slogan” (text passages in which the interviewees expressed their opinion about the slogan) identified comments on the effect and suggestions for improvement. Overall, it became apparent that the majority considered the slogan to be appealing and motivating. Suggestions for improvement were related to giving the slogan a different name.

Under the main category “*overall communication strategy,”* three sub-categories could be formed. “Contribution to promote physical activity on site” (statements on how the overall communication strategy helps to put the employees into motion) showed the characteristics of “can contribute,” “cannot contribute,” and “no assessment possible.” In terms of “can contribute,” stakeholders gave the estimation that the communication strategy can create initial access to physical activity. Nevertheless, they also expressed skepticism concerning this or could not give an estimation. Concerning the “amount of information” (feedback on the quantity of information provided in the project), stakeholders stated that it was suitable. Concerning the “general appraisal” (statements concerning the overall impression of the communication strategy), stakeholders reported that the communication strategy was essentially well done.

“*Yes. Well, I think it could be a part of this. It could never, I think never, would it be sufficient on its own, but it could contribute to somehow to pick-up people. Because this page is trying to appeal to people and to send the message: It doesn't matter what you do. What is important is to keep moving. To do your body some good. And yes, that a variety of components dealing with movement are addressed. And there it can make a contribution. It can be a small part of something bigger.” (network partner, female)*

### Facilitating factors for and barriers to successful communication of physical activity measures in the cross-company network

Regarding research question (3), two main categories were defined: *(f) facilitating factors and (g) barriers*. In addition, the overarching category *(h) coronavirus* was identified. Table 5 illustrates the main categories, their definitions as well as the sub-categories, and characteristics.

**Table 5:** Coding system: facilitation factors and barriers.

**Main category (definition)**	**Sub-categories**	**Characteristics**
Facilitating Factors	Alternative use of media	• Digital
(Factors that can promote the communication about physical activity measures in a cross-company network)		• Analogous
	Creation of personal contact possibilities	• Low-threshold measures on site • Establishment of an e-mail distribution list with employees • Info points • Company visits
	Communication management	• Regular information • Individual support • Variety of channels • To live the messages communicated • Cross-company exchange
	Public relation activities	• Visibility • Highlights • Moving images • Talking about the positive actions undertaken • Use of the press
	Participation of stakeholders	• Multipliers • Employees • Management • Direct supervisors • Exercise providers
	Exhaustion of existing access paths	• High-traffic locations • Established information systems
Barriers (Factors that can hinder the communication and awareness of physical activity measures in a cross-company network)	People Lack of information transfer Priority of other topics Time	
Coronavirus (Communicable disease; the impact of the coronavirus pandemic on the project and the communication of physical activity measures on site)		

Within the main category “*facilitating factors,”* six sub-categories were defined: “alternative use of media,” “creation of personal contact possibilities,” “communication management,” “public relations activities,” “participation of stakeholders,” and “exhaustion of existing access paths.”

The sub-category of “alternative media use” describes channels that were seen as promotive for the communication and the announcement of physical activity measures that extend beyond the channels used to date within the project. Thereby, digital and analogous media were mentioned. In the context of digital media, stakeholders advocated for creating a cross-company digital platform, which facilitates the communication between the employees and not only presents information about the measures. Furthermore, they indicated that the use of social media, apps, blogs, intranet, and QR-codes could be beneficial. Concerning analogous media, stakeholders suggested fliers combined with giveaways, an intensified use of posters, and the creation of a project newspaper as well as the use of information boards.

The “creation of personal contact opportunities” meant opportunities for personal exchange with employees on site. Most stakeholders proposed carrying out low-threshold measures (e.g., inaugural event, action days) to engage in conversation with the employees. On the other hand, the implementation of an e-mail distribution list for all interested employees, the establishment of info spots on site where people can seek information about the project, and company visits could be the opportunities to promote communication.

The aspects of “communication management” (aspects that should be taken into account in the context of communication planning) covered a broad spectrum, within which the regularity of information about measures was identified as one essential factor. Stakeholders also felt that it was beneficial to take employees and companies by the hand and provide individual support. Likewise, regular cross-company exchange was recommended. In addition, the use of a variety of communication channels, taking into account the different prerequisites in the companies, was mentioned. Finally, it was highlighted how important it is to live the messages communicated.

In the sub-category “public relation activities” (what can be done to be perceived by employees), stakeholders reported that initiators/actors of such a project should be consistently visible for the employees; for example, in person on site or *via* video. Besides, it was proposed to set unusual, accompanying highlights such as coffee rounds or early bird actions. In order to arouse the curiosity of the employees, the use of moving images was also suggested. Further recommendations related to using the press and regularly talking about the positive actions undertaken.

In the sub-category of “participation of various stakeholders” (individuals who can be beneficial for communicating physical activity measures in a cross-company network), most stakeholders indicated that multipliers in the companies play an important role for promoting the topic internally. When interviewees named more specific actors, they mentioned the increased involvement of employees, management, or direct supervisors. The exercise providers themselves could potentially publicize the measures during their courses and thus increase their awareness.

The final sub-category identified was “exhaustion of existing access paths” (usage of channels or locations that are already established on site). In this context, the stakeholders recommended a connection to platforms that are already used for communication in the companies. Besides, high-traffic locations such as cafeterias, parking facilities, meeting rooms, or main entrances could be specifically used to place information.

“*So, I have to repeat myself there because, at the end of the day, you have to tap into various channels. Because, as I said, the companies differ so much, you know. They are from different branches, they have different employees, office jobs, and then some are on the machines. It is very, very, very different. And in such cases, you have to try and use all channels.” (network partner, male)*

“*What comes to mind quite clearly for me is to have an impetus which really supports the issue.” (network partner, female)*

In the main category “*barriers,”* a total of four sub-categories could be categorized: “people,” “lack of information transfer,” “priority of other topics,” and “time.”

The first sub-category of “people” was defined as the dependence on various actors in making measures known. Thus, both the recipient him/herself and the commitment of the person responsible for communication in the companies can represent a barrier from the stakeholders' perspective. Besides, stakeholders reported that the “lack of information dissemination” can also be a barrier for making measures known on site. It described the fact that information is not passed on in the companies. Stakeholders also cited “priority of other topics” and “time” as additional barriers. Thus, they mentioned that other issues may have higher priority for project partners than the communication of physical activity measures, while a lack of time during the workday may also influence the communication of physical activity measures in this setting.

“*And then there is another difference. Do I just pass this on without a comment. Or do I at least write something about it, or do I use other paths of communication internally, inside the company. By saying: I will address several executives, so that they take it up. Or, let's say, draw attention to it in other ways in my company. Well, it depends so very, very much on the individual people. And that is, I just see the difficulty there.” (network partner, female)*

“*Coronavirus”* could be identified as another main category. Overall, it became clear that the pandemic situation was an overarching factor influencing the communication and awareness of physical activity measures within the cross-company network. The stakeholders mentioned that due to the pandemic situation many measures and project management activities had to be implemented digitally, personal contact on site was restricted and many companies also had to struggle with the new pandemic situation.

“*And if Corona is already a difficult situation. And a lot is being restructured in the company. Then there is the possibility that someone might not deal with it quickly enough, or be able to deal with it, that creates problems too. And then maybe the employees are not on site regularly. Then it will get really difficult.” (exercise provider, female)*

## Discussion

The present study aimed to evaluate a communication strategy for promoting physical activity in a cross-company network. Furthermore, it was aimed to derive findings for a successful communication of measures promoting physical activity. Our results show that a variety of factors must be considered when communicating physical activity measures in a cross-company network, and that it is important to engage in an appropriate communication management. It is noticeable that reaching the target group depends on more than simply welldesigned media or messages; rather, factors like an interpersonal communication should be explicitly taken into account. Beyond this, the major influence of organizational circumstances on the successful communication of measures becomes clear.

As shown in Figures 2, 3, the usage of the digital communication channels within the project was subjected to major fluctuations. Given the high number of employees on site (about 2000), potentially many more visitors could have used the website or newsletter subscription. Therewith, the usage of the digital communication channels fell short of expectations. Comparing Figure 2, Table 3, it was noticeable that an increase in the number of users was mostly associated with the announcement of new measures *via* the distribution list of the participating companies and the dispatch of newsletters. The same applies to the distribution list of the operator, albeit to a much lesser extent. The fact that the e-mail distribution list of the participating companies had a better effect than the distribution list of the operator could indicate that distribution to a broad unknown mass is not automatically more adjuvant. Nevertheless, it also opened the chance to reach other companies. Essentially, this procedure seemed to have achieved the goal of calling attention to new measures presented on the website. In times when no communication activities were carried out (e.g., weeks 58–61, 64–67), the number of users remained at a low level. Vacation periods also seemed to have an impact on website usage, with hardly any users over the turn of the year and the first summer vacations during the project (weeks 13–18, 38–40). However, it should be noted that fewer measures for the cross-company network were usually implemented at these times. A very closely timed mailing of newsletters—as was done in the second year of the project (starting from week 46)—caused the number of users to repeatedly increase but did not seem to lead to more frequent use of the website over time. Why the number of users was extraordinarily high—especially in week 47—leaves room for discussion. During this time, there was a partial lockdown to control the epidemic in Germany and fitness studios were closed. The project was offering a digital back pain prevention course at this time and based on the registration number information seemed to get around in one company in particular. Likewise, one measure was promoted by a fitness studio *via* Instagram. It cannot be explained by a reliable cause–effect relationship. Comparing Figure 3, Table 3, the number of newsletter subscribers could also be linked to the dispatch of new information *via* the distribution lists of the participating companies. Why there was a sharp drop in subscribers in week 42 remains questionable. During this period, students were involved in the project who possibly enrolled and quickly disenrolled for the newsletter.

Considering the website users, parallels may be drawn to the use of web-based interventions. As mentioned above, low usage data and high attrition rates are a common problem (27, 28). To counteract this problem, blended interventions—i.e., interventions that combine digital and analog approaches—seem to be more effective (58, 59). A number of studies (60, 61) have also reported that regular reminders and personal contact provided can also have a positive impact. Comparable to the importance of blended approaches in the context of web-based interventions, such serration may also play a decisive role in the context of publicizing and using digital information platforms or newsletters, as applied in the present project. Prospective regular visitation or registration could be promoted during (kick-off) events on site. This recommendation could be supported by Stassen et al. (62), who have shown that initial face-to-face contact can be helpful to log on to a web-based platform. As mentioned in the literature (25), the strength of one-on-one conversation is a very high target group specificity and interactivity. Furthermore, one-on-one conversation can achieve a very high depth of information, credibility, and clarity, which underlines the importance of their usage (25). Combined with regular reminders *via* company representatives and repetitive personal contacts on site, the existence of such information channels could be advertised on a regular basis. To avoid ignorance, information should be sent out regularly but not too closely timed. This would be in line with Geraghty (63), who reported that multiple e-mails can also be counter-productive. Drawing on earlier work by Budde (64), it is also important to maintain such information platforms continuously and create incentives for visits. Finally, the problem of media disruption that Bonfadelli and Friemel (39) mention should be highlighted. According to the researchers (39), the use of websites always requires other channels in a campaign that draw attention to the website.

The fact that an increase in website users and newsletter subscriptions was closely related to the chosen communication paths showed that they can be a useful way to draw attention to measures in a cross-company network. This can also be supported by the qualitative results of our study in which stakeholders considered these communication paths as a useful way to spread information. In the literature, too, the possibility of reaching a specific target group *via* mailings is rated high (25). Generally, emails seem to be a good way to communicate information about physical activity (26). Nevertheless, it became clear how important it is to keep one's eyes on possible interface problems with this type of communication paths and avoid information flooding. The latter notion corresponds with previous findings (49) where circulars were critically considered in times of information overload. Besides, the potential of mailings to increase the perceived relevance of the topic is low (25). However, coupled with our various communication channels, good requirements were created for informing employees about measures in a cross-company network. Thereby, each channel implicated its own advantages and disadvantages, which should be considered in advance and in the context of the framework conditions in the company (e.g., what work is performed and how can the employee's best be reached). Baumann et al. (25) also recommend to compile an individual media mix for each communication project, as the potentials of the individual channels differ significantly from one another and the content, objectives, and target group in projects can vary. Further literature (39) also highlights that no general recommendations can be made about the selection of channels. It must also be pointed out that the effort and costs of channels differ greatly (25). While costs and effort for websites tend to be high, mailings are significantly cheaper (25), so that decisions must also be made based on budget within the framework of communication projects.

However, for media design and message conception, no general recommendations could be derived from our findings. Following McGuire's first three steps in the model of persuasion (54), we cannot say conclusively whether our messages were well designed to persuade. The possibility of exposing employees to the messages was severely limited by the pandemic and there was a variety of different opinions by the stakeholders about the message's attention generation and understanding. The significance of coherent messages—especially in the context of mass media campaigns—has been highlighted in the literature (65). Williamson et al. (66) recommend that physical activity messages for adults should be brief and framed positively (e.g., addressing the benefits). In WHP projects and especially in such cross-company networks, one should possibly ascribe more attention to the strategic approach of information brokerage than the development of the design and messages. As Budde (64) has noted, a positive public image and advertising are insufficient for the success of WHP and their disclosure. In this context, the author emphasizes the importance of a systematic and strategic approach of communication (64), which is also mentioned by further researchers (44, 67). Nevertheless, the design aspect should not be completely disregarded. As Nöhämmer (68) showed in an earlier qualitative study, information in the context of health promotion should show appreciation for the employees, which is also achieved through an appealing design. In this context, Bergeron et al. (26) also emphasize the importance of a high quality of information transmitted.

Our identified facilitating factors and barriers toward successful communication also showed parallels with previous research. The use of alternative communication channels beyond newsletters, websites, and posters was cited as a key factor in terms of a successful communication within a cross-company network. This is also described as a success factor in literature (39, 64, 69).^2^ According to Bergeron et al. (26), various modalities and different channels that convey the messages can enhance the impact of a campaign. Furthermore, our findings indicate that face-to-face contact should be ensured. Several studies have emphasized that conversational communication holds strong significance (21, 64, 68, 70). As Walter et al. (21) and Baumann et al. (25) have highlighted, there is a higher probability of reaching addressees. Our results indicate that in this context, low-threshold events and activities that enable personal conversation with employees provide an opportunity. A study by Stummer et al. (49) also identified such symbolic events as being conducive to successful communication. Thereby, our results suggest that the exchange should also be made possible within the target group. This could potentially be realized by providing a corresponding function within the information platforms, which was a recommendation by the stakeholders. According to the literature (25), the strength of such online forums can be seen in a very high target group specificity and interactivity, which in turn could have a beneficial effect on the communication process.

Moreover, our results suggest considering existing access paths and good public relations. As also described in the literature (6), public relations' activities are one central plank in the context of WHP. Overall, the entire communication procedure should be integrated into a management, as already mentioned above. Among others, this takes into account the regularity of information, which is also recommended by the literature (68, 69). Thereby, communication of WHP measures should always consider the organizational context. As Stummer et al. (49) have noted, organizational conditions can have a significant influence on the success of communication and the timing of communication is essential. Thus, in the context of health marketing, the literature (46) also refers to the appraisal of organizational prerequisites within the planning process. In a cross-company context—as in our study—this cannot always be realized but should be considered as a possible influencing factor. Basically, some disadvantages can be mentioned regarding communication processes in cross-company networks (compared to communication in single companies). First of all, a higher planning effort for the communication strategy can be assumed. Companies from a wide range of sectors can be represented in cross-company networks. Often—depending on the sector—the everyday work differs greatly. Besides, the target group can be very heterogeneous. In addition, it is more difficult to involve the target group in the conception, since there are significantly more employees to deal with. There is also a challenge in specifically adapting the communication process to the needs of the individual company. However, if reliable contact persons are available in the companies and good internal structures for passing on information have already been created, cross-company networks also offer the opportunity to address health messages to a broad mass.

Our findings also support previous research concerning the importance of different stakeholders in the communication of WHP measures. In particular, engaged multipliers represent an important facilitating factor. Several studies (21, 44, 64) have reported the importance of a timely involvement of various multipliers, whereby managers and work councils in particular are mentioned in this context. As we have seen in our study, the focus was not so strongly placed on the profession of the multiplier in the company, but rather the importance of the personal commitment and the willingness and motivation to address the issue, particularly when it comes to promoting behavioral change and accessing difficult-to-reach target groups. Wäsche (44) emphasizes the important role of informal actors in the company (e.g., respected colleagues) to transmit health-related information.

In many cases, stakeholders reported that people could also act as a barrier in the communication of measures. Baumann and Hastall (71) also highlight the influence of people regarding the success of health communication. As it is also known from the literature (72, 73), successful WHP is related to the support of the management level. Furthermore, it should not be forgotten that measures are still voluntary and people cannot be forced to participate (6). Finally, a lack of time and the priority of other topics—which are known challenges in the context of WHP, especially in smaller companies (6, 74, 75)—were named as barriers for successful communication.

Overall, the implementation of the communication strategy was strongly influenced by the coronavirus pandemic. This may also be one reason for the low usage data of the website and the newsletter. Throughout the course of the project, the pandemic situation made it almost impossible to get in personal contact with the employees. Among others, a major inaugural event on site during which the project was due to be widely publicized had to be canceled. Since no on-site events could be conducted, the banner and business cards were not used. Additionally, the posters were distributed much less frequently, partly due to the fact that the technology park was less frequently visited due to the pandemic. As the literature points out (25, 76), posters offer a high degree of clarity and can again help to increase the perceived relevance of the topic. Communication options were therefore also limited by the fact that many employees worked in their home offices. The qualitative data support these statements, as stakeholders also saw the pandemic as a strong influencing factor. Besides, current research (77) confirms that there were difficulties in reaching target groups due to the pandemic circumstances. As Bonfadelli (33) has noted, insufficient time to reach the target group is one factor that can limit the success of communication campaigns. This may have also been true for the present project.

Home office working will most likely continue to play a major role in the future (77). This is attached to new challenges for WHP measures and their communication. Therefore, hybrid approaches will continue to be relevant in the next years, which once again requires effective communication management and close exchange with the companies. However, the literature (78) indicates that e-health tools can be supportive in WHP, including when it is about information and communication. Finally, our results support the assumption that the cross-linked use of instruments with a dialog-oriented focus is significant for communication as Walter et al. (21) postulated in their model for the systematization of communication tools for WHP. Nevertheless, additional research is necessary to confirm these results, especially in the context of cross-company networks.

### Strengths and limitations

The results of our study offer insights into the design of communication strategies to promote physical activity in cross-company networks and allow drawing conclusions for the general WHP praxis. Thus, this study makes an important contribution to the transfer of science and practice as it captures relevant questions from praxis. Thereby, the serration of qualitative and quantitative data enabled a comprehensive evaluation of the communication strategy implemented. The monitoring of the usage data provided an initial insight into the applied communication strategy at the output level. Afterward, the results of the monitoring contributed to the development of the interview guide. The interviews themselves enabled a deeper insight into the communication strategy. Overall, serration of data thus made it possible to develop a better overall interpretation of the communication strategy.

However, it is necessary to acknowledge some limitations. Regarding the planning conception and development of the communication strategy, it could be critically noted that no employees or project partners were involved, although this is recommended in the literature (25, 39, 79) (e.g., *via* participatory measures as survey or workshop). A lack of time at the beginning of the project did not allow for this. Second, the evaluation and interpretation of the qualitative results are based on a weak empirical foundation with only 14 participants with heterogeneous backgrounds. There could also have been a response bias in the evaluation of the communication strategy since the interviewees were all part of the project and there is a barely assessable extent to which the results of the interviews may appear to be socially desirable. Further research is required to generalize the qualitative findings concerning the facilitators and barriers. As with all interventions in WHP, the present study was a complex intervention in a complex setting, and it was strongly influenced by the coronavirus pandemic. Therefore, the results should be interpreted conservatively. Besides, the limits of the meaningfulness of the usage data must be pointed out. The number of users does not provide evidence about the real number of users. It is also possible that people visited the website more than once (e.g., from different devices), which would be associated with an even lower number of total users. Also, we did not distinguish between the number of users and the number of views and there is also no socio-demographic data available to gain more insights concerning the usage data. Finally, reference must be made to the general depth of our analysis. The KomRueBer project was a model project that did not allow to contribute to an evaluation of effectiveness. The overall study focused a descriptive approach. Therefore, it was not the aim of the present study to conduct an evaluation of effectiveness concerning the communication strategy. Rather, the present paper must be seen as part of a clear process evaluation, which is a strength at the same time. It provides valuable information in terms of quality development in the context of communication processes in WHP and contributes to the output level in the overall context of the impact model-based evaluation of the KomRueBer study.

## Conclusion

Communication is an important factor that should always be taken into account when planning WHP projects. This mixed-methods study revealed which factors may have an influence on the successful communication of physical activity measures in the context of WHP in cross-company networks. Thereby, the importance of active management of the communication process became clear. Our results provide initial suggestions for successful campaign management in future cross-company networks. As part of the project, we primarily focused on developing professional messages and design during the development of the communication strategy. However, the results also show the strong importance that should be attached to the strategic planning of the entire communication process. Reaching the target group requires a systematic approach, not only in individual companies but also in cross-company networks featuring companies from different sectors and with different sizes and requirements. In any case, media should be appealing and messages appreciative. Nevertheless, we recommend not investing excessive time in this regard, but rather trying to create spaces for personal contact opportunities with people on site. We also recommend involving the target group in the planning process from beginning. In addition, the structural conditions in the companies should be recorded promptly, for example, as part of introductory working groups. From our perspective, a communication strategy should be included into every WHP project as it creates initial access to the topic. To sum up, the results of our study provide valuable information on how communication strategies for physical activity in cross-company networks can be designed. Thus, the study addresses a relevant problem from practice—namely, reaching the target group for health promotion measures. The evaluation approach with focus on the output level is a valuable basis for subsequent effectiveness studies and takes on an important role concerning quality development in the context of WHP.

## Data availability statement

The datasets used and analyzed during the current study are available from the corresponding author on reasonable request.

## Ethics statement

The studies involving human participants were reviewed and approved by Ethics Committee of the German Sport University Cologne (Reference Number: 068/2020). The patients/participants provided their written informed consent to participate in this study.

## Author contributions

CH and AS: conceptualization and methodology. CH: analysis, visualization, and writing—original draft. AS: resources, project administration, funding acquisition, and writing—review and editing. Both authors have read and approved the final manuscript.

## Footnotes

^1^ https://www.destatis.de/DE/Themen/Arbeit/Arbeitsmarkt/Erwerbstaetigkeit/Tabellen/insgesamt.html

^2^ https://www.cdc.gov/workplacehealthpromotion/planning/communications.html
